# Interoceptive sensibility tunes risk-taking behaviour when body-related stimuli come into play

**DOI:** 10.1038/s41598-019-39061-0

**Published:** 2019-02-20

**Authors:** Gerardo Salvato, Gabriele De Maio, Gabriella Bottini

**Affiliations:** 1 Department of Brain and Behavioral Sciences, University of Pavia, Pavia, Italy; 2 Cognitive Neuropsychology Centre, ASST “Grande Ospedale Metropolitano” Niguarda, Milano, Italy; 3 NeuroMI, Milan Centre for Neuroscience, Milan, Italy

## Abstract

In everyday life, we continuously make decisions, assuming the risk by making choices on material possessions or our body. Bodily signals may support the decision-making process, informing us about possible outcomes. Sensibility for such internal bodily changes influences the way we perceive the environment, and it can boost the body-related stimuli processing. Thus, the question arises of whether the individual sensibility to interoceptive signals modulates decision-making in the presence of biological stimuli. To test this hypothesis, we administered 50 healthy subjects with the Balloon Analogue Risk Task, in which participants were required to inflate a virtual balloon, and a modified version of it, in which they inflated a virtual body. We found that interoceptive sensibility predicted risk-taking behaviour only in the presence of body-related stimuli. Our results provided new evidence on the role of interoceptive sensibility in complex cognitive functions, such as risk-taking behaviour, which impacts the way we act within our society.

## Introduction

Humans interact within the environment, integrating information coming from inside and outside of the body. Where the influence of external signals on cognition has mainly been investigated, the impact of the sense for the internal bodily changes (interoception) is a relatively new topic of investigation. Interoception has been mainly measured using objective behavioural tests, such as the heartbeat perception tasks (interoceptive accuracy), throughout self-evaluated assessment interviews/questionnaires (interoceptive sensibility) or by estimating the confidence-accuracy correspondence (interoceptive awareness)^1^. It is now increasingly recognised that these three dimensions intermingle with cognitive functions along a continuum, from a fine-graded/implicit to a coarser/explicit tuning of interaction^2^. Amongst higher-level cognitive functions, decision-making under risk has attracted particular interest concerning the role of interoception. According to the Somatic Marker Hypothesis, interoceptive information (e.g., elevated heart rate and sweating) would play a pivotal role in risky decisions, warning individuals of possible outcomes^3,4^.

Evidence on the influence of interoception on decision-making is divergent mainly due to the different approaches used to investigate such a relationship^5–9^. It is important to notice that, so far, only object-related stimuli have been employed in risk-taking behaviour paradigms (e.g., cards, balloons). However, in real-world decision-making under risk does not only concern objects or material possessions. In our everyday life, we also take risks with our bodies: one need only think, for example, of extreme sports, or even more simply, riding our bikes for a few meters without a helmet.

Recent evidence has shown that interoceptive accuracy and sensibility enhanced the cognitive processing of body-related stimuli^10,11^. Thus, the question arises of whether risk-taking behaviour would be modulated by interoception, manipulating the nature of the stimuli to be processed. Here, we aimed to explore whether interoceptive sensibility would predict risk-taking behaviour in the presence of body-related stimuli. To this end, we instructed 50 healthy subjects to complete a modified version of the Balloon Analogue Risk Task (BART)^12^, in which participants were required to inflate a virtual body (Body task). Participants were also administered with a control task, in which they were required to inflate a virtual balloon (Balloon task). We hypothesised that higher interoceptive sensibility would lead to a decrease in risk-seeking behaviour in the task in which a body silhouette was presented.

## Results

We modelled a multivariate multiple regression using the adjusted number of pumps for the Balloon and Body tasks (*risk index*)^12,13^ as dependent variables, and we used the interoceptive sensibility scores as a covariate (predictor). The two dependent variables were normally distributed, whereas the predictor was log10-transformed to fit a normal distribution. All of the regression assumptions were met. To deal with outliers affecting the values of the estimated regression coefficients, we performed a casewise diagnostic on standardised residuals. We excluded from the subsequent analyses one outlier (outside ± three standard deviations) (*SD* residual = 3.3). Results showed that the interoceptive sensibility scores negatively predicted the number of pumps in the Body task (*b* = −0.34; *t*_(48)_ = −2.4; *p* = 0.018; *R*^2^ = 0.21; *F*_(1,48)_ = 6.4; *p* = 0.004). Participants with higher interoceptive sensibility made fewer pumps at the body task, showing a more conservative behaviour when a body-related stimulus came into play. Contrarily, interoceptive sensibility did not predict the Balloon task outcome (*b* = −0.09; *t*_(48)_ = −0.6; *p* = 0.530; *R*^2^ = 0.09; *F*_(1,48)_ = 2.2; *p* = 0.123).

To better frame this finding, we supplemented the frequentist analysis with a Bayesian approach^14,15^ performing two separate linear regressions between the adjusted number of pumps in the Body and Balloon tasks and the interoceptive sensibility measure to test whether there was evidence for supporting the alternative hypothesis against the null hypothesis. For the relationship between interoceptive sensibility and the adjusted number of pumps in the Body task, we found strong evidence for the alternative against the null hypothesis (*BF*_10_ = 11.8), whereas for the Balloon task there was anecdotal evidence for the alternative against the null hypothesis (*BF*_10_ = 0.6).

## Discussion

We continuously evaluate the risks/benefits ratio of possible scenarios to make a decision. Some of these decisions may involve material possessions, whereas others involve the body. The present study provided new evidence of the role of interoceptive sensibility, which selectively modulates risk-taking behaviour in the presence of body-related stimuli. In a nutshell, participants with higher scores on the Body Awareness subscale of the Body Perception Questionnaire (BPQ)^16^ were more risk-averse when inflating a virtual body compared to a balloon.

Evidence for the role of interoceptive accuracy or sensibility in decision-making is conflicting. Dunn and colleagues^7^ have demonstrated that bodily signals during the execution of a decision-making task influenced decisions more strongly as interoceptive ability increased. Werner and colleagues^6^ have demonstrated that individuals with good heartbeat perception chose significantly fewer disadvantageous and more advantageous options in the Iowa Gambling Task, a monetary risk-taking paradigm. Later, Werner and colleagues^5^ failed to find such a relationship using the same task. It has also been demonstrated that interoceptive sensibility to bodily signals selectively predicted aversion to losses in one’s choices independently from risk-taking behaviour *per se*^9^. In another study investigating the interoceptive ability of traders, it has been found that traders were better able to perceive their heartbeats than matched controls. Moreover, the interoceptive ability of traders predicted their relative profitability and how long they survived in the financial markets^8^. Lastly, in a more recent work, no relationship was found between interoceptive sensibility and probability discounting, a measure of impulsivity in decision-making^17^. These discrepant findings may have been produced by a variety of experimental paradigms and stimuli used to measure risk-taking behaviour.

Hunt and colleagues^18^ have demonstrated that the performance at the BART (using balloons as stimuli) did not correlate with physiological markers, such as heart rate responsivity and skin conductance responses. These results offer a possible explanation for the present study, in which we did not find a relationship between the Balloon task and interoceptive sensibility. One might speculate that participants may have used different strategies to perform the two tasks. In the case of object-related stimuli, their performance would have relied on visual properties of the stimulus, whereas, in the case of body-related stimuli, they may have relied on internal bodily signals. Nevertheless, we did not collect any qualitative data on the approaches used by our participants, and therefore cannot provide evidence supporting strategy differences.

Body-related compared to object-related stimuli engage distinctive neurofunctional processing. The brain perceives visual stimuli concerning the body in a domain-specific manner^19–21^ within selective specialised neural networks^19,22,23^. Brain regions such as the insula and the parietal cortex^24–30^ may represent a good candidate, subserving the interplay between the sense for the internal bodily changes and bodily cognition, as they have been linked to both interoception^26,31,32^ and processing of body-related stimuli^24,25^.

One may also speculate that sensibility to bodily signals may have enhanced sensibility to body-related stimuli, as they may rely on shared neural networks. Our group has recently demonstrated that highly sensible participants to their bodily signals showed a stronger memory-guidance of spatial attention towards body compared to face and chair pictures^11^. Furthermore, Ronchi and colleagues^10^ have shown that interoceptive accuracy enhanced visual processing for the body compared to scrambled-body images when shown to the participants in synchrony with their heartbeats. Further studies are needed to better clarify such complex interplay between interoception and cognition at both behavioural and neurofunctional levels.

## Materials and Methods

### Participants

Fifty healthy participants (25 females, 25 males; age range 20–28, *M* = 23.5, *SD* = 2.1; years of education *M* = 14.8, *SD* = 1.9) attended the experiment. All were students from the University of Pavia, native Italian speakers, and had a normal or corrected-to-normal vision with no previous history of mental or neurological illness. In accordance with the Declaration of Helsinki (BMJ 1991; 302: 1194), all the experimental procedures were approved by the Ethical Committee of the Department of Brain and Behavioral Sciences, University of Pavia. Informed consent was obtained before participation in the experiment.

### Task

#### Balloon and Body Analogue Risk Tasks

Each participant was administered with two tasks: the BART^12^ and a modified version of it. In the BART, a balloon was presented at the centre of the screen, along with a balloon pump, a button labelled “*Collect $$$*”, a permanent display labelled “*Total Earned*” indicating the earned money, and a second display listing the money earned on the last balloon labelled the “*Last Balloon*”. The subject was asked to click on the pump. Each pump caused a size increase in the balloon (about 0.125 in. [0.3 cm] in all directions), accompanied with a pump sound effect. Additionally, five cents were banked in a temporary reserve (not indicated to the subject). When a balloon was pumped to its explosion point, a “pop” sound effect was generated, causing the loss of all banked money. The subjects were instructed that they could stop pumping and click the “*Collect $$$*” button at any time, transferring all temporary money to the permanent bank. A slot machine payoff sound effect played as the subject clicked on the “*Collect $$$*” button. After the explosion or the money collection, the balloon disappeared, and a new balloon appeared until a total of 90 balloons. The maximum number of pumps of a single trial is pseudo-randomly chosen in a range, which is determined by the balloon’s colour (orange life range = 1–8 pumps, yellow life range = 1–32 pumps, blue life range = 1–128). The three different colours had the purpose of generating an experience-based risk-taking task. They were presented over three experimental blocks: Block 1 contained the three balloon variants; Block 2 contained the orange and yellow balloons; and Block 3 contained the yellow and blue balloons. Participants were not instructed regarding this difference. Rather, they learned during the task that some stimuli had a certain chance of explosion, depending on their colours. Such a learning effect would lead participants to increase their risk-taking behaviour over the three blocks linearly. Typically, participants pumped more on the last experimental block when only blue balloons were present, having learned that those had the lowest probability of exploding compared to other balloon colours.

**Figure 1:**
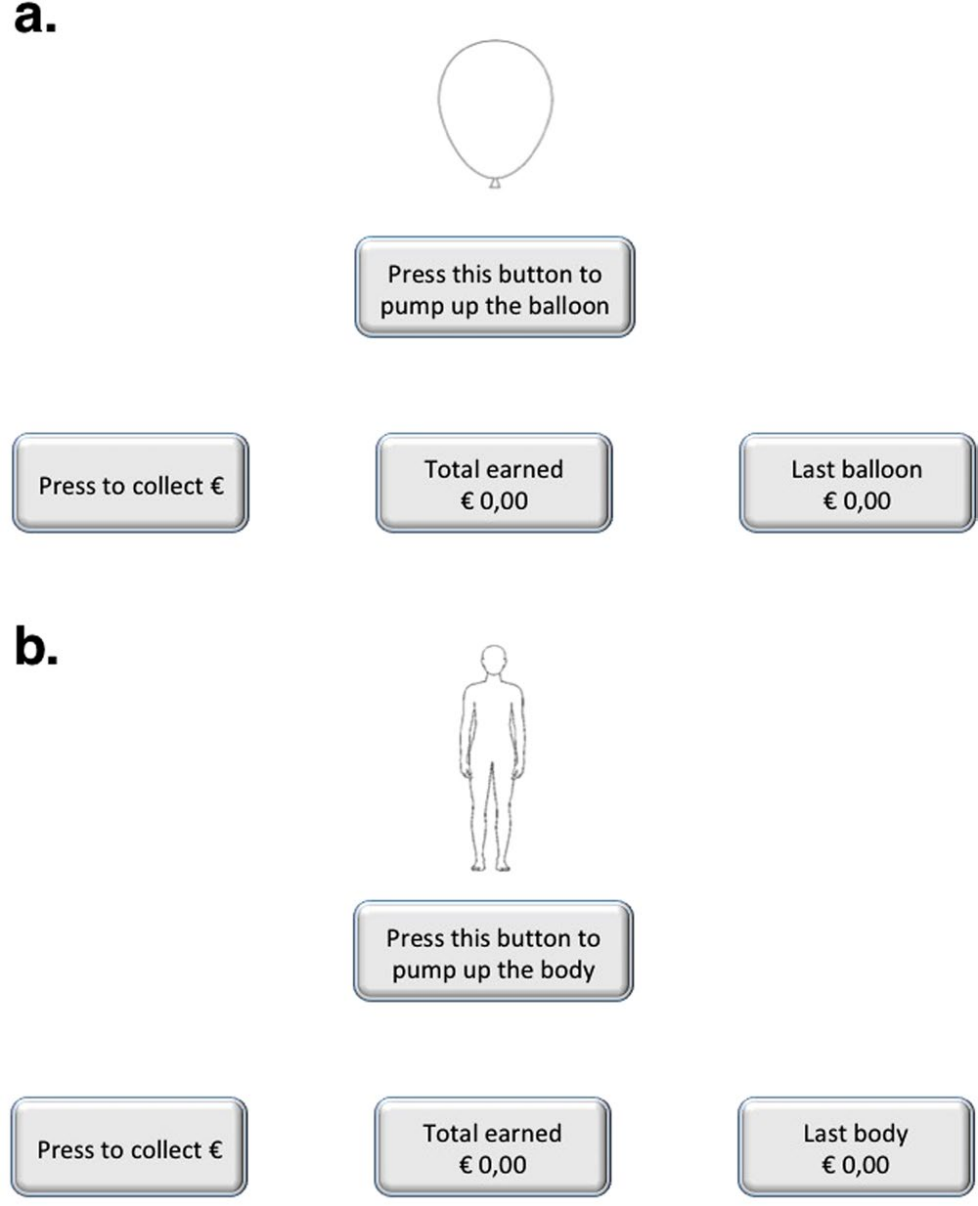
Tasks. Panel “a” shows an example for the Balloon task, and panel “b” shows an example for the Body task.

In the current study, we implemented the BART and replaced the balloon with a body silhouette (see Fig. 1). As in the case of balloons, each pump increased the stimulus size (about 0.125 in. [0.3 cm] in all directions), and was accompanied by a pump sound effect. Importantly, the human configuration of the silhouette remained plausible throughout inflation. The experimental design was equal to the original BART for the Balloon and Body tasks. Thus, the two tasks had the same number of trials, blocks, and coloured shapes. Each participant performed the two tasks in a randomised order within the group. The instructions were the same as the original version of the BART^12^. The administration order of the two tasks was randomised across participants. Participants did not receive money gained in the tasks, but course credit for their participation.

As a measure of risk-seeking behaviour, we used an adjusted value, defined as the average number of pumps on the blue stimuli, excluding those that exploded (i.e., the average number of pumps on each balloon/body prior to money collection) because the number of pumps was necessarily constrained on stimulus that exploded, thereby limiting between-subjects variability in the absolute averages^12^. As the blue stimulus allowed the widest range of the possible number of pumps and therefore was likely to capture the greatest amount of individual variability in task performance, the adjusted number of pumps on this stimulus across blocks served as the primary dependent measure (*risk index*)^12,13^. Following this procedure, we discarded 60 trials for each participant.

#### Interoceptive Sensibility

Participants were also administered with the Body Perception Questionnaire (BPQ)^16^, a well-known measure of interoceptive sensibility^1,33–36^. The BPQ is a self-report 122-item that requires to indicate the individual level of awareness for several bodily sensations using a five-point scale ranging from “never” to “always”. In line with previous works^1,33–36^ and the aim of the present study, here we took into account the first 45 items of the questionnaire constituting the Body Awareness subscale. The Body Awareness subscale has been widely used in previous research (for a review see)^37^. Our group has also provided further evidence on the use of the Body Awareness subscale that selectively predicted top-down attention to bodies^11^. Furthermore, the validity of this subscale has also been corroborated by neuroimaging studies, demonstrating its association with the insulae grey matter density and neural activity in healthy participants^26,38^.

### Apparatus

The tasks were programmed and administered using PEBLportable^39^. A personal computer controlled the stimulus displays and collected the behavioural responses. The stimuli were displayed on a 24-inch monitor with a resolution of 1028 by 768 pixels and a 60-Hz refresh rate.
